# COVID-19 Social Distancing Measures and Loneliness among Older Adults

**DOI:** 10.1093/geroni/igaa057.3438

**Published:** 2020-12-16

**Authors:** Eun Young Choi, Matthew Farina, Qiao Wu, Jennifer Ailshire

**Affiliations:** 1 University of Southern California, Los Angeles, Los Angeles, California, United States; 2 University of Texas at Austin, Austin, Texas, United States

## Abstract

In response to the COVID-19 pandemic, older adults are advised to follow social distancing measures to prevent infection. However, such measures may increase the risk of loneliness. The current study aimed to investigate (1) whether social distancing measures, particularly limiting close social interactions, are associated with loneliness among older adults, and (2) whether the association between social distancing measures and loneliness is moderated by sociodemographic characteristics. Data were from the fourth wave (April 29 to May 26, 2020) of the nationally representative Understanding America Study (UAS) COVID-19 Survey. We used data on adults 50 years or older (N = 3,283). Multivariate logistic regression models of loneliness were examined to test the independent effects of social distancing measures and their interaction with sociodemographic characteristics on loneliness. Four indicators of social distancing measures were considered: (a) avoiding public spaces, gatherings, or crowds, (b) canceling or postponing social activities, (c) social visits, (d) close contact (within 6 feet) with others. Cancelling or postponing social activities and avoiding close contact with other people were associated with 36% and 41% greater odds of loneliness, respectively. Furthermore, males and non-Hispanic Whites who had no close contact with others had a significantly greater probability of reporting loneliness than those who had contact. Our findings emphasize the heterogeneous nature of COVID-19 related experiences across subpopulations of older adults and call special attention to vulnerable groups that may be more impacted by the challenge of COVID-19 social distancing.

